# Chlorpyrifos Affects Phenotypic Outcomes in a Model of Mammalian Neurodevelopment: Critical Stages Targeting Differentiation in PC12 Cells

**DOI:** 10.1289/ehp.8750

**Published:** 2005-12-29

**Authors:** Ruth R. Jameson, Frederic J. Seidler, Dan Qiao, Theodore A. Slotkin

**Affiliations:** Department of Pharmacology and Cancer Biology, Duke University Medical Center, Durham, North Carolina, USA

**Keywords:** acetylcholine, brain development, catecholamines, chlorpyrifos, DNA synthesis, organophosphate insecticides, PC12 cells

## Abstract

The organophosphate insecticide chlorpyrifos (CPF) adversely affects mammalian brain development through multiple mechanisms. To determine if CPF directly affects neuronal cell replication and phenotypic fate, and to identify the vulnerable stages of differentiation, we exposed PC12 cells, a model for mammalian neurodevelopment, to CPF concentrations spanning the threshold for cholinesterase inhibition (5–50 μM) and conducted evaluations during mitosis and in early and mid-differentiation. In undifferentiated cells, exposure to 5 μM CPF for 1–3 days reduced DNA synthesis significantly without eliciting cytotoxicity. At the same time, CPF increased the expression of tyrosine hydroxylase (TH), the enzymatic marker for the catecholamine phenotype, without affecting choline acetyltransferase (ChAT), the corresponding marker for the cholinergic phenotype. Upon exposure to nerve growth factor (NGF), PC12 cells developed neuritic projections in association with vastly increased TH and ChAT expression accompanying differentiation into the two phenotypes. CPF exposure begun at the start of differentiation significantly reduced ChAT but not TH activity. In contrast, when CPF was added in mid-differentiation (4 days of NGF pretreatment), ChAT was unaffected and TH was increased slightly. Thus, CPF exerts stage-specific effects, reducing DNA synthesis in the undifferentiated state, impairing development of the cholinergic phenotype at the start of differentiation, and promoting expression of the catecholaminergic phenotype both in undifferentiated and differentiated cells. CPF administration *in vivo* produces deficits in the number of neurons and cholinergic function, and because we were able to reproduce these effects *in vitro*, our results suggest that CPF directly influences the phenotypic fate of neuronal precursors.

It is increasingly evident that organophosphate insecticides affect mammalian brain development through a variety of mechanisms. In animal studies or *in vitro* models of neurodevelopment, chlorpyrifos (CPF; *O*,*O*-diethyl *O*-[3,5,6-trichloro-2-pyridyl ester]phosphorothioate), the most widely applied insecticide, has direct and indirect effects on neural cell replication and differentiation, resulting in immediate and delayed-onset changes in synaptogenesis (Barone et al. 2000; Casida and Quistad 2004; Gupta 2004; Pope 1999; Slotkin 1999, 2004; Slotkin et al. 2001), neurotransmitter release (Aldridge et al. 2005; Bloomquist et al. 2002; Dam et al. 1999a; Liu and Pope 1998), expression of neurotransmitter receptors (Chaudhuri et al. 1993; Dam et al. 1999b; Huff and Abou-Donia 1995; Katz et al. 1997; Richardson and Chambers 2005), and intracellular signaling (Huff et al. 2001; Meyer et al. 2004a; Schuh et al. 2002), over and above the consequences of cholinesterase inhibition. Because of this diversity, the critical period for the effects of CPF exposure depends highly upon the maturational stage of each brain region as well as the specific neurotransmitter, producing a wide window of vulnerability with shifting regional and cellular targets as development proceeds (Pope 1999; Rice and Barone 2000; Slotkin 2004). Indeed, CPF can even exert simultaneous, opposite effects on axonal and dendritic growth (Howard et al. 2005).

The disparate nature of CPF effects on brain development, combined with potential impact on the maternal–fetal unit or general aspects of fetal or neonatal growth and development, renders it especially difficult to identify specific underlying mechanisms from *in vivo* studies or to discern why specific developmental stages or neurotransmitter systems might be especially targeted by CPF. Accordingly, recent attention has focused on *in vitro* models, including neuronotypic and gliotypic cells as well as primary cultures of mixed neurons and glia (Garcia et al. 2001; Howard et al. 2005; Olivier et al. 2001; Sachana et al. 2001; Schuh et al. 2002; Song et al. 1998). PC12, a transformed neuronotypic cell line, provides one of the most useful model systems for evaluations of developmental neurotoxicants (Crumpton et al. 2000; Das and Barone 1999; Fujita et al. 1989; Madhok and Sharp 1992; Qiao et al. 2001, 2005; Song et al. 1998; Teng and Greene 1994). As immature neuronal precursors, these cell maintain mitotic activity in culture (whereas primary neurons lose that ability), enabling assessments of adverse effects on DNA synthesis and cell replication (Qiao et al. 2001, 2003; Song et al. 1998). Upon addition of nerve growth factor (NGF), PC12 cells begin to differentiate and develop axonal projections and electrical excitability (Das and Barone 1999; Fujita et al. 1989; Teng and Greene 1994), whereas mitotic activity gradually declines over a period of about 10 days (Qiao et al. 2001, 2003; Song et al. 1998). Critical for their use in modeling the developmental neurotoxicity of CPF, PC12 cells differentiate specifically into two phenotypes, cholinergic and catecholaminergic (Fujita et al. 1989; Teng and Greene 1994). Studies of CPF effects on brain development *in vivo* indicate high susceptibility of these two transmitter systems, but with disparate patterns. Cholinergic systems are among the most sensitive, showing both immediate and lasting damage when CPF exposure occurs during periods of rapid cell replication and differentiation (Barone et al. 2000; Pope 1999; Rice and Barone 2000; Slotkin 2004; Vidair 2004); in contrast, the initial effects on catecholamine systems appear to be promotional (Dam et al. 1999b), although long-term deficits in function eventually appear (Aldridge et al. 2005; Slotkin et al. 2002) as part of the pattern of late-developing, widespread disruption of synaptic connectivity in multiple neurotransmitter systems (Barone et al. 2000; Pope 1999; Rice and Barone 2000; Slotkin 2004; Vidair 2004).

In this study we used the PC12 model to address several key aspects of the developmental neurotoxicity of CPF. Does CPF have disparate effects on neuronal development during phases of cell replication as opposed to differentiation? Does CPF alter the ability of developing neurons to express a specific neurotransmitter phenotype, and if so, at what stage of maturation does this occur? Do these changes occur at CPF concentrations below those necessary for effects on viability? To answer these questions, we evaluated PC12 cells in the undifferentiated state (CPF without NGF), at the initiation of differentiation (inclusion of CPF simultaneously with the addition of NGF), and at mid-differentiation (CPF added after several days of NGF pretreatment), evaluating cell viability, DNA synthesis associated with cell replication, and enzymatic markers that characterize the cholinergic or catecholaminergic phenotype: choline acetyltransferase (ChAT) and tyrosine hydroxylase (TH), respectively. Because CPF exposure can affect the number of cells, cell growth, or neuritic extension (Das and Barone 1999; Song et al. 1998), values were determined relative to cell number (per unit DNA) as well as to cell protein. Assessments were conducted at 5–50 μM CPF (1.7–17 μg/mL), concentrations bracketing the threshold for cholinesterase inhibition (Das and Barone 1999) and for adverse effects on cell number (Song et al. 1998). Finally, to determine whether nonorganophosphate cholinesterase inhibitors could elicit similar effects, we evaluated the effects of physostigmine compared with CPF.

## Materials and Methods

### PC12 cell culture and treatments.

As described previously (Crumpton et al. 2000; Qiao et al. 2003; Song et al. 1998), PC12 cells (1721-CRL; American Type Culture Collection, Rockville, MD) were grown in RPMI-1640 medium (Invitrogen, Carlsbad, CA) supplemented with 10% inactivated horse serum (Sigma Chemical Co., St. Louis, MO), 5% fetal bovine serum (Sigma), and 50 μg/mL penicillin–streptomycin (Invitrogen); cells were incubated with 7.5% CO_2_ at 37°C, and the medium was changed every 2 days. Because of the clonal instability of the PC12 cell line (Fujita et al. 1989), the experiments were performed on cells that had undergone fewer than five passages, and studies were repeated several times with different batches of cells. For studies in the undifferentiated state, 3–6 × 10^6^ cells were seeded onto 100-mm poly-l-lysine–coated plates, and 24 hr later the medium was changed to include 5 or 50 μM CPF (purity, 98%; Chem Service, West Chester, PA). CPF was dissolved in dimethyl sulfoxide (DMSO), achieving final DMSO concentrations of 0.1–1% in the culture medium, and the corresponding control samples contained equivalent DMSO concentrations. Preliminary studies were conducted to verify that these concentrations of DMSO had no effect on the measured parameters in PC12 cells (data not shown), in agreement with earlier work (Qiao et al. 2001, 2003; Song et al. 1998).

For studies during differentiation, 3.5 × 10^6^ cells were seeded; 24 hr later the medium was changed to include 50 ng/mL NGF (Sigma), and over the ensuing week, each culture was examined under a microscope to verify the outgrowth of neurites. CPF or physostigmine (Sigma) was added either simultaneously with the addition of NGF or after a 4-day NGF pretreatment.

### DNA synthesis.

To initiate the measurement of DNA synthesis, the medium was changed to include 1 μCi/mL [^3^H]thymidine (specific activity, 2 Ci/mmol; GE Healthcare, Piscataway, NJ) along with the continued inclusion of CPF or DMSO vehicle. After 1 hr, the medium was aspirated and cells were harvested in 3.5 mL ice-cold water. Duplicate aliquots of each sample were treated with 10% trichloroacetic acid and sedimented at 1,000 × *g* for 15 min to precipitate macromolecules. The resulting pellet was washed once with additional trichloroacetic acid and then with 75% ethanol. The final pellet was hydrolyzed with 1 M KOH overnight at 37°C and neutralized with 6 M HCl, and the DNA was precipitated with ice-cold 5% trichloroacetic acid and resedimented. The supernatant solution, comprising solubilized RNA and protein, was discarded. The DNA-containing pellet was hydrolyzed in 5% trichloroacetic acid for 15 min at 90°C and resedimented, and an aliquot of the supernatant solution was counted for radiolabel. Another aliquot was assayed for DNA spectrophotometrically by absorbance at 260 nm. Previous work has demonstrated quantitative recovery of DNA by these techniques (Bell et al. 1986; Slotkin et al. 1984). Incorporation values were corrected to the amount of DNA present in each culture to provide an index of macromolecule synthesis per cell.

### Enzyme activities.

Cells were harvested as described above and were disrupted by homogenization in a ground-glass homogenizer fitted with a ground-glass pestle, using a buffer consisting of 154 mM NaCl and 10 mM sodium-potassium phosphate (pH 7.4). An aliquot was withdrawn for measurement of protein (Smith et al. 1985).

ChAT assays (Lau et al. 1988) were conducted with 40 μg of sample protein in 60 μL of a buffer consisting of 60 mM sodium phosphate (pH 7.9), 200 mM NaCl, 20 mM choline chloride, 17 mM MgCl_2_, 1 mM EDTA, 0.2% Triton X-100, 0.12 mM physostigmine (Sigma), and 0.6 mg/mL bovine serum albumin (Sigma), containing a final concentration of 50 μM [^14^C]acetyl-coenzyme A (specific activity of 44 mCi/mmol, diluted with unlabeled compound to 6.7 mCi/mmol; PerkinElmer Life Sciences, Boston, MA). Blanks contained homogenization buffer instead of the tissue homogenate. Samples were preincubated for 15 min on ice, transferred to a 37°C water bath for 30 min, and the reaction terminated by placing the samples on ice. Labeled acetylcholine was then extracted and counted in a liquid scintillation counter, and the activity was determined relative to DNA or protein.

TH activity was measured using [^14^C]tyrosine as a substrate and trapping the evolved ^14^CO_2_ after coupled decarboxylation with DOPA decarboxylase (Lau et al. 1988; Waymire et al. 1971). Homogenates were sedimented at 26,000 × g for 10 min to remove storage vesicles containing catecholamines, which interfere with TH activity, and assays were conducted with 100 μL aliquots of the supernatant solution in a total volume of 550 μL. Each assay contained final concentrations of 910 μM FeSO_4_, 55 μM unlabeled l-tyrosine (Sigma), 9.1 μM pyridoxal phosphate (Sigma), 36 μM β-mercaptoethanol, and 180 μM 2-amino-6,7-dimethyl-4-hydroxy-5,6,7,8-tetrahydropteridine HCl (Sigma), all in a buffer of 180 mM sodium acetate and 1.8 mM sodium phosphate (pH 6.1). Each assay contained 0.5 μCi of generally labeled [^14^C]tyrosine (specific activity, 438 mCi/mmol; Sigma) as substrate, and blanks contained buffer in place of the homogenate.

### Viability.

Cell viability was assessed in separate cultures. The medium was changed to include trypan blue (Sigma; 1 vol per 2.5 vol of medium), and cells were examined for staining under 400× magnification, counting an average of 100 cells per field in four different fields per culture.

### Data analysis.

Data are presented as means and SEs. For each type of study, treatment differences were first evaluated with a global analysis of variance (ANOVA; data log-transformed whenever variance was heterogeneous) incorporating all variables: cell batch number, CPF concentration, time, and the presence or absence of NGF. Based on the interactions of CPF with the other factors, data were then subdivided for lower-order tests, followed by Fisher’s protected least significant difference to establish individual values that differed from the corresponding controls. Significance was assumed at the level of *p* < 0.05 for main effects; however, for interactions at *p* < 0.1, we also examined whether lower-order main effects were detectable after subdivision of the interactive variables (Snedecor and Cochran 1967). In the initial test, the results did not vary among the different batches of cells, so results across the different batches were combined for presentation, and the indicated number of samples in each experiment reflects the total number of cultures.

## Results

### Undifferentiated cells.

**Figure 1:**
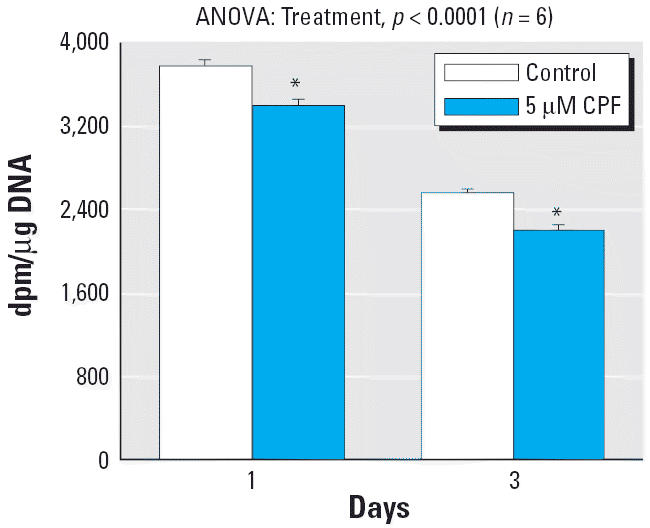
Effects of CPF treatment on DNA synthesis in undifferentiated PC12 cells. Data are means and SEs. ANOVA across both time points appears at the top of the panel. *Significantly different from the corresponding control.

**Figure 2:**
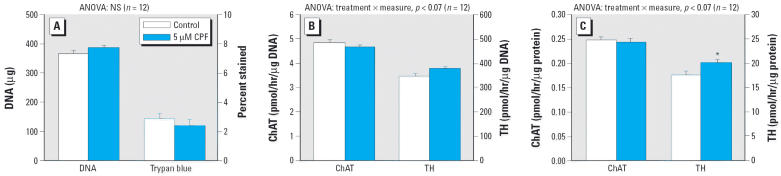
Effects of a 3-day CPF treatment on undifferentiated PC12 cells. NS, not significant. (*A*) Cell number (DNA content) and percentage of nonviable cells (trypan blue staining). (*B*) ChAT and TH activity per cell (i.e., per microgram DNA). (*C*) ChAT and TH activity per microgram protein. Data are means and SEs. *Significantly different from the corresponding control. Across both types of enzyme activity measures [per microgram DNA, per microgram protein, (*B*) and (*C*) taken together], ANOVA also indicated a significant treatment × measure interaction (*p* < 0.02) with no significant effect on ChAT (*p* > 0.3) but a significant increase in TH (*p* < 0.05).

Earlier work demonstrated that CPF causes immediate decrements in DNA synthesis in undifferentiated PC12 cells (Qiao et al. 2001; Song et al. 1998). Because the present study evaluated longer-term effects, we measured [^3^H]thymidine incorporation after CPF exposures of 1 and 3 days (Figure 1). At 5 μM, the threshold concentration for immediate inhibition of DNA synthesis (Song et al. 1998), CPF elicited a significant reduction in [^3^H]thymidine incorporation that was sustained through the 3 days of treatment. Nevertheless, the total number of cells remained within normal bounds, and there was no decrease in viability as assessed with trypan blue staining (Figure 2A); similarly, indices of overall cell growth (protein:DNA ratio) or cell surface area (membrane protein/total protein) were not significantly affected (data not shown). Across both measurements (per microgram DNA, per microgram protein; Figure 2, B and C taken together) of the neurotransmitter-related enzymes, evaluation of ChAT and TH indicated a significant CPF × enzyme interaction (*p* < 0.02), reflecting a preferential increase in TH (*p* < 0.05) without an effect on ChAT. For the index of activity per cell (Figure 2B), there was no significant effect on either enzyme, whereas activity per unit protein showed a small but significant increase in TH (Figure 2C).

### CPF exposure commencing at differentiation.

**Figure 3:**
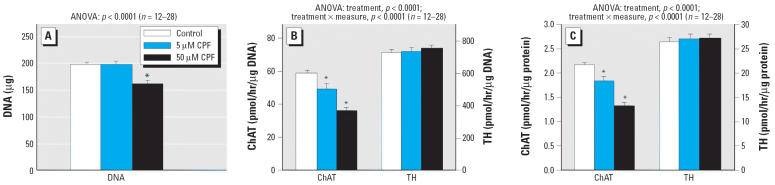
Effects of CPF treatment on differentiating PC12 cells; CPF was added simultaneously with NGF and differentiation was carried out for a 7-day period. (*A*) Cell number (DNA content). (*B*) ChAT and TH activity per cell (i.e., per microgram DNA). (*C*) ChAT and TH activity per microgram protein. Data are means and SEs. *Significantly different from the corresponding control. Across both types of enzyme activity measures (per microgram DNA, per microgram protein), ANOVA also indicated a significant main treatment effect (*p* < 0.0001) and a treatment × measure interaction (*p* < 0.0001) with a significant decrease in ChAT (*p* < 0.0001) but no significant effect on TH (*p* > 0.8).

When 5 μM CPF was added simultaneously with NGF, there was no change in total DNA at the end of a 1-week exposure, but raising the concentration to 50 μM produced a significant shortfall (Figure 3A); earlier work showed only a small (3%) decrease in viability after even longer exposures to much higher CPF concentrations (Song et al. 1998). By itself, NGF-induced differentiation elicited massive increments in ChAT and TH activity relative to undifferentiated cells (compare scales in Figure 2B,C vs. Figure 3B,C). In stark contrast to the lack of effect of CPF on ChAT in undifferentiated cells, the addition of CPF at the start of differentiation resulted in a profound deficit in ChAT, discernible at either 5 or 50 μM CPF for activity expressed either per microgram DNA (Figure 3B) or per microgram protein (Figure 3C). TH showed small increments at either concentration that did not achieve statistical significance but that were also not distinguishable from the significant increments found in undifferentiated cells (no CPF × ±NGF interaction).

### CPF added after the initiation of differentiation.

**Figure 4:**
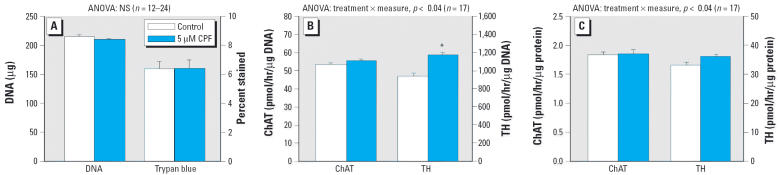
Effects of CPF treatment after the initiation of differentiation in cells pretreated with NGF for 4 days before the addition of CPF and with incubations continued for 3 additional days. NS, not significant. (*A*) Cell number (DNA content) and percentage of nonviable cells (trypan blue staining). (*B*) ChAT and TH activity per cell (i.e. per microgram DNA). (*C*) ChAT and TH activity per microgram protein. Data are means and SEs. *Significantly different from the corresponding control. Across both types of enzyme activity measures (per microgram DNA, per microgram protein), ANOVA also indicated a significant treatment × measure interaction (*p* < 0.006) with no significant effect on ChAT (*p* > 0.3) but a significant increase in TH (*p* < 0.04).

When cells underwent 4 days of NGF-induced differentiation before the addition of CPF, quite different results were obtained for effects on ChAT and TH, but not for general indices of cellular development. By 1 week after the initiation of differentiation (7 days after the start of NGF, exposure to 5 μM CPF for the final 3 days), there was a small decrement in DNA content at the margin of statistical significance (*p* < 0.06) and no change in viability as assessed with trypan blue staining (Figure 4A). ChAT activity per cell was unaffected by CPF treatment, whereas the corresponding measure for TH showed a significant increase (Figure 4B). Activity per unit protein showed the same trend, although for this measure the TH effect was at the margin of significance (*p* < 0.07) (Figure 4C). The lack of effect on ChAT was entirely separable from the significant effect seen when CPF was added simultaneously with NGF (*p* < 0.003 for CPF × treatment paradigm). However, the increment in TH was indistinguishable from the smaller, nonsignificant promotional effect seen with the simultaneous NGF + CPF paradigm, or from the significant increment in undifferentiated cells (*p* < 0.01 for the main effect of CPF, without a significant interaction of CPF × treatment paradigm).

### Effects of physostigmine.

**Figure 5:**
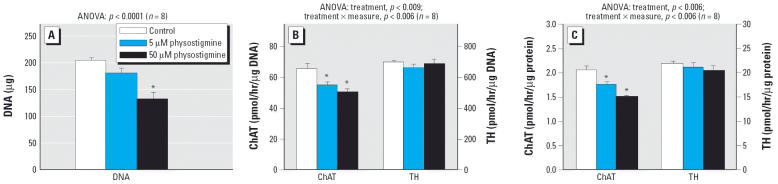
Effects of physostigmine treatment on differentiating PC12 cells. Physostigmine was added simultaneously with NGF and differentiation was carried out for a 7-day period. (*A*) Cell number (DNA content). (*B*) ChAT and TH activity per cell (i.e., per microgram DNA). (*C*) ChAT and TH activity per microgram protein. Data are means and SEs. *Significantly different from the corresponding control. Across both types of enzyme activity measures (per microgram DNA, per microgram protein), ANOVA also indicated a significant main treatment effect (*p* < 0.002) and a treatment × measure interaction (*p* < 0.0001) with a significant decrease in ChAT (*p* < 0.0001) but no significant effect on TH (*p* > 0.4).

To contrast the effects of CPF with a nonorganophosphate cholinesterase inhibitor, we performed parallel studies in differentiating cells exposed to a carbamate, physostigmine. Compared with CPF, physostigmine elicited much greater deficits in DNA but slightly smaller effects on ChAT. At 50 μM, physostigmine evoked loss of more than one-third of the cells (Figure 5A) while reducing ChAT about 20% relative to DNA (Figure 5B) or 25% relative to protein (Figure 5C). Physostigmine had no net effect on TH activity relative to either DNA or protein (Figure 5B,C).

## Discussion

In earlier work, CPF was shown to inhibit cell acquisition and neurite outgrowth in PC12 cells (Das and Barone 1999; Qiao et al. 2001; Song et al. 1998), mimicking some of the primary effects of this agent on the developing brain *in vivo* (Barone et al. 2000; Pope 1999; Rice and Barone 2000; Slotkin 2004). Results of the present study indicate that, at the same concentrations, CPF also has direct effects on differentiation that determine the phenotypic fate of developing neuronotypic cells. Specifically, the cholinergic phenotype is vulnerable at the earliest stage of differentiation, the transition from cell replication to specialization, and the CPF effects are seen in the absence of significant cell loss or cytotoxicity. Indeed, the period in which ChAT was susceptible to CPF is specifically the stage in which PC12 cells shift away from mitosis and toward differentiation into the two phenotypes, in association with greatly increased levels of neurotransmitter synthetic enzymes (Kalisch et al. 2002). As shown here, the effects on ChAT expression display a clearly delineated critical period, with virtually no effect either in undifferentiated cells or in later stages of differentiation. By way of contrast, the slight promotional effect on the catecholaminergic phenotype was seen in both undifferentiated and differentiated cells, suggesting an entirely separate mechanism. Importantly, the same dichotomy for the two phenotypes is seen in the developing brain after CPF exposure *in vivo* (Barone et al. 2000; Dam et al. 1999b; Pope 1999; Rice and Barone 2000; Slotkin 2004; Vidair 2004). Indeed, similar changes may occur in peripheral cholinergic and catecholaminergic pathways, where CPF exposure in neonatal rats enhances β-adrenergic effects at the expense of muscarinic cholinergic actions (Meyer et al. 2004b; Slotkin et al. 2005). Our findings thus provide an underlying mechanism for the differential effects of CPF on the diverse neuronal cell populations contained within each brain region, superimposed on more general aspects of cell loss and impaired axonogenesis and synaptogenesis (Slotkin 1999, 2004).

The lower concentration used here, 5 μM, lies within the range of estimated household childhood exposure to CPF (Gurunathan et al. 1998) and of CPF concentrations in fetal meconium (Ostrea et al. 2002) but is below the threshold for cholinesterase inhibition (Das and Barone 1999); the *in vivo* CPF metabolite, CPF oxon, is the active agent that produces irreversible anticholinesterase actions and is three orders of magnitude more potent than CPF itself in inhibiting cholinesterase (Das and Barone 1999). Like most neuronotypic cells, PC12 cells lack cytochrome P450 activity, which is required for conversion to the oxon (Mapoles et al. 1993), so inhibition is obtained only when the concentration is raised above the 5 μM value (Das and Barone 1999). Given that the symptoms of organophosphate intoxication do not emerge until cholinesterase activity is reduced by > 70% (Clegg and van Gemert 1999), the impact on neuronal development of the cholinergic phenotype is likely to be seen even with exposures that typically go unrecognized. Indeed, the adverse effect on early differentiation into cholinergic neurons appears to be among the most sensitive effects, independent of the more general inhibition of cell replication or axonogenesis.

At 5 μM CPF, although we found significant inhibition of DNA synthesis on undifferentiated cells, there was no cumulative deficit in cell number as assessed by the DNA content, whereas at higher concentrations, these deficits become significant (Song et al. 1998). Evidently, the reduction in DNA synthesis is either offset by the small, nonsignificant improvement in cell viability (reduced trypan blue staining), or alternatively, other phases of the cell cycle are rate limiting for replication, such that a slight slowing of DNA synthesis has no impact on the number of cells. Nevertheless, with the initiation of NGF-induced differentiation, the same concentration had a profound impact on ChAT. As further evidence for a complete separation of these two effects, in the differentiating cells, 5 μM CPF exerted its adverse effects on the cholinergic phenotype without affecting DNA content or cell viability; raising the concentration to 50 μM compromised cell acquisition, but then there was an even more profound effect on ChAT (20% deficit in DNA, 40% deficit in ChAT:DNA), again consistent with specificity toward expression of the cholinergic phenotype. The targeting of cholinergic differentiation is also distinct from effects of CPF on axonogenesis. When we pretreated the cells with NGF, followed by a subsequent CPF exposure, the adverse effect on ChAT was no longer evident; in contrast, the same sequence enhances the ability of CPF to inhibit neurite outgrowth (Das and Barone 1999). Finally, the ability of CPF to impair differentiation into the cholinergic phenotype does not represent selective cytotoxicity. We saw no evidence for decreased viability, nor did we see an equivalent increase in the TH:DNA ratio as would have been expected from the selective loss of cholinergic cells and sparing of catecholaminergic cells. Similarly, in primary neuronal/glial cultures, CPF reduces ChAT expression without comparable cytotoxicity (Monnet-Tschudi et al. 2000; Zurich et al. 2004).

The results for TH, the marker for appearance of the catecholaminergic phenotype, confirm that CPF does not simply impede cell differentiation as a whole. Rather than showing a decrease, TH activity was induced when CPF was applied to cells either in an undifferentiated state or after the onset of differentiation. This suggests that CPF alters basal TH activity through mechanisms entirely separable from its impact on differentiation into the cholinergic phenotype. Although our studies do not address the molecular mechanisms underlying the dissimilar impact on cholinergic and catecholaminergic phenotypes, there are a number of likely candidates. ChAT and TH expression are controlled through separate transcriptional promoter pathways, comprising cAMP-dependent processes (adenylyl cyclase, protein kinase A, CREB, AP-1) as well as trophic factors such as brain derived neurotrophic factor, ciliary neurotrophic factor, repressor element-1 silencing transcription factor/neuron restrictive silencer factor, and bone morphogenetic protein-9 (Lewis-Tuffin et al. 2004; Lopez-Coviella et al. 2000; Shimojo and Hersh 2004; Slonimsky et al. 2003). There is abundant evidence for CPF’s targeting of the cAMP signaling cascade and its associated downstream transcriptional elements (Casida and Quistad 2004; Crumpton et al. 2000; Meyer et al. 2003; Schuh et al. 2002; Song et al. 1997), and our results suggest the need to explore the impact on other transcriptional factors specific to neuronal differentiation into the cholinergic and catecholaminergic phenotypes; it is quite likely that the targeting of ChAT versus TH results from activation or suppression of some of these factors or their downstream effectors at specific developmental stages.

The viability and differentiation of PC12 cells require the inclusion of fetal calf and horse serum and their associated proteins (Crumpton et al. 2000; Das and Barone 1999; Fujita et al. 1989; Madhok and Sharp 1992; Qiao et al. 2001, 2005; Song et al. 1998; Teng and Greene 1994). Organophosphates, including CPF, are highly bound to albumin, thereby lowering the bioeffective CPF concentration both *in vivo* and in culture (Braeckman et al. 1983; Qiao et al. 2001). Earlier, we showed that serum proteins protect PC12 cells from the adverse effects of CPF (Qiao et al. 2001), and accordingly, their inclusion in the present study means that the suppressed emergence of the cholinergic phenotype is exerted at CPF concentrations well below those actually added to the cultures. The same factors are likely to be important for CPF effects on brain development *in vivo*. The fetus and neonate have lower concentrations of serum albumin than does the adult, so the bioeffective concentration of CPF is higher than at the corresponding level in the mother (Thom et al. 1967; Yaffe and Stern 1976). Furthermore, because albumin concentrations correlate with somatic growth (Thom et al. 1967), low-birth-weight infants may be especially susceptible to the effects of CPF. The “Barker Hypothesis” relates low birth weight to the emergence of cardiovascular and metabolic diseases later in life (Barker 2003), and thus our results suggest that this relationship may equally apply to adverse neurodevelopmental consequences after exposure to environmental toxicants.

One final question is whether these effects are specific to CPF or rather are shared by other organophosphate insecticides or by nonorganophosphate cholinesterase inhibitors such as carbamates. Like CPF, diazinon inhibits DNA synthesis in undifferentiated PC12 cells, whereas physostigmine, a carbamate, is less effective (Qiao et al. 2001). Despite that, in cells undergoing NGF-induced differentiation, we found greater cell loss with physostigmine (35% reduction in DNA) than with CPF (20% reduction), implying that the carbamate derivative is more generally deleterious in long-term exposures of differentiating cells. In part, this likely reflects the fact that physostigmine does not bind as strongly to serum proteins (Whelpton and Hurst 1990), so its effective concentration in the culture system may be considerably higher than that of CPF. Over and above effects on cell number, the fact that physostigmine shares the ability to inhibit differentiation into the cholinergic phenotype strongly suggests a common contributing factor. The first likely explanation is cholinesterase inhibition. Indeed, a transition occurs *in vivo* for the effects of CPF on cell acquisition and differentiation, where actions are initially independent of cholinergic hyperstimulation but become increasingly cholinesterase-related as differentiation proceeds (Slotkin 1999, 2004). However, we also detected effects of CPF on ChAT at low concentrations that did not impair cell acquisition and below the threshold for cholinesterase inhibition; furthermore, cholinesterase knockout mice show normal development of cholinergic projections, including unchanged expression of ChAT (Volpicelli-Daley et al. 2003). It is therefore possible that organophosphates and carbamates share additional mechanisms other than cholinesterase inhibition that nevertheless contribute to developmental neurotoxicity targeted to the cholinergic phenotype. These complex relationships also are apparent in other test systems. In aggregating cultures of primary neurons and glia, CPF inhibits the emergence of the cholinergic phenotype much more than does parathion, despite the fact that the latter is more systemically toxic and more potent toward cholinesterase inhibition, and physostigmine is less effective (Monnet-Tschudi et al. 2000; Zurich et al. 2004). Again, our findings are entirely compatible: although physostigmine reduced ChAT:DNA 20%, CPF produced a much greater effect (40% deficit) without a comparable degree of cell loss.

In conclusion, CPF exerts stage-specific effects on neuronal development, reducing DNA synthesis in the undifferentiated state, impairing emergence of the cholinergic phenotype at the start of differentiation, and promoting expression of the catecholaminergic phenotype in both undifferentiated and differentiated cells. Because similar effects are seen *in vivo* after fetal or neonatal CPF exposures at levels below the threshold for systemic effects or even for cholinesterase inhibition (Barone et al. 2000; Casida and Quistad 2004; Gupta 2004; Pope 1999; Slotkin 1999, 2004; Slotkin et al. 2001), it is likely that CPF has a direct effect on neuronal differentiation, specifically compromising the development of the cholinergic phenotype. In turn, these alterations may relate mechanistically to deficient cholinergic synaptic function and later-developing neurobehavioral deficits (Barone et al. 2000; Pope 1999; Rice and Barone 2000; Slotkin 2004). Finally, given the relative facility with which multiple end points of cell acquisition, cytotoxicity, and phenotypic differentiation can be evaluated in the PC12 system, we suggest that this model can be especially useful for the rapid screening of neurodevelopmental outcomes of related or disparate environmental neurotoxicants.
